# Learnability of Ultrasound-Guided Locoregional Anesthesia for Carotid Endarterectomy

**DOI:** 10.3390/jcm13247557

**Published:** 2024-12-12

**Authors:** Benjamin Seybold, Nils Gaier, Andreas Ofenloch, Dittmar Boeckler, Armin Kalenka, Mascha O. Fiedler-Kalenka

**Affiliations:** 1 Department of Anesthesiology, Heidelberg University Hospital, Medical Faculty, University of Heidelberg, 69120 Heidelberg, Germany; armin.kalenka@med.uni-heidelberg.de (A.K.); mascha.fiedler-kalenka@med.uni-heidelberg.de (M.O.F.-K.); 2 Merck KGaA, 64293 Darmstadt, Germany; 3 Department of Vascular Surgery, District Hospital Bergstrasse, 64646 Heppenheim, Germany; andreas.ofenloch@med.uni-heidelberg.de; 4 Department of Vascular and Endovascular Surgery, Heidelberg University Hospital, Medical Faculty, University Heidelberg, 69120 Heidelberg, Germany; dittmar.boeckler@med.uni-heidelberg.de; 5 District Hospital Bergstrasse, 64646 Heppenheim, Germany

**Keywords:** locoregional anesthesia, learning curve, cervical plexus blockade, ultrasound, carotid endarterectomy

## Abstract

**Background/Objectives**: There is an ongoing debate about the most advantageous anesthesia technique for carotid endarterectomy (CEA). From an anesthesiologic perspective, locoregional anesthesia (LRA) appears to offer significant benefits. However, the learning curve and complication rates for anesthesiologists newly performing ultrasound-guided LRA for CEA remain unclear and are to be examined in greater detail in this study. **Methods:** This retrospective, single-center study included all consecutive LRA administrations for CEA following the introduction of this procedure at a district hospital in Germany from November 2013 to November 2017. Nine board-certified anesthesiologists, initially inexperienced in LRA for CEA but with prior experience in other ultrasound-guided peripheral nerve blocks (PNBs), received theoretical training and supervision during their first six combined deep and superficial cervical plexus blocks under ultrasound guidance. The primary endpoint was the incidence of insufficient block quality, indicated by pain and restlessness or the additional need for analgesics. Secondary endpoints included LRA-associated complications. Patients were divided into four groups based on the number of previously performed LRA procedures by the attending anesthesiologist. **Results:** In 83 patients, LRA was performed by initially inexperienced anesthesiologists. Group A (patients managed by anesthesiologists performing their 1st to 3rd cervical plexus blockades) included 21 patients, Group B (blockades 4–6) included 12 patients, Group C (blockades 7–9) included 9 patients, and Group D (≥10 blockades) included 41 patients, respectively. The overall complication rate was 22% (18/83). Insufficient block quality occurred in 18.1% of patients (15/83), resulting in three conversions to general anesthesia (3.6%). Additional complications included dysphagia (n = 2) and Horner’s syndrome (n = 1). The incidence of insufficient block quality was significantly reduced (*p* = 0.008) after performing the first three blockades. **Conclusions:** Ultrasound-guided cervical plexus block for CEA appears to be a rapidly learnable anesthesia technique for anesthesiologists experienced in other ultrasound-guided PNBs, with a low risk of complications. After three supervised blockades, the failure rate of LRA decreases significantly.

## 1. Introduction

There is an ongoing debate about whether carotid endarterectomy (CEA) should be performed under locoregional anesthesia (LRA) or general anesthesia (GA). The largest randomized controlled trial (RCT), the General Anaesthesia versus Local Anaesthesia (GALA) trial, which included 3526 participants, found no significant difference in the rates of perioperative death, stroke, or myocardial infarction between GA (4.8%) and LRA (4.5%) [1]. However, a large meta-analysis found that LRA was associated with significantly shorter operation times, lower rates of perioperative stroke, fewer cardiac complications, and lower mortality in the 25 observational studies included [2]. Nevertheless, each of the six included RCTs failed to demonstrate statistically significant differences in any endpoints [2]. Some authors believe that these RCTs lacked statistical power [3]. Recent subgroup analyses from two studies suggest that LRA may indeed be associated with a slightly lower risk of stroke and mortality compared to carotid endarterectomy under GA [4,5].

The European Society for Vascular Surgery Management Guidelines of Atherosclerotic Carotid and Vertebral Artery Disease recommends that the choice of anesthesia (locoregional or general) should be made at the discretion of the surgeon and anesthesiologist performing the procedure, taking into account local experience, patient preferences, and the preferred antiplatelet strategy [6]. Consequently, in the absence of a medical contraindication for either approach, anesthesiologists and surgeons should mutually consider each others’ preferences. Locoregional anesthesia should be performed under ultrasound guidance, as this can enhance the safety of this technique by providing visualization of cervical anatomical structures during infiltration [7]. However, the learnability of ultrasound-guided regional anesthesia remains poorly studied, and the number of blocks required to achieve proficiency is still a matter of debate [8].

Therefore, the implementation of CEA, performed by surgeons experienced in this procedure at a district hospital in Germany, provided an opportunity to retrospectively evaluate the learning curves of anesthesiologists inexperienced in ultrasound-guided cervical plexus blockades. Here, we report on the learnability and complication rates of ultrasound-guided LRA for CEA in a supervised setting for board-certified anesthesiologists newly performing LRA in this anatomical region. The primary endpoint was the incidence of insufficient block quality indicated by pain, restlessness, or the requirement for additional local or systemic anesthetics.

## 2. Materials and Methods

### 2.1. Study Design and Population

This is a retrospective, single-center study conducted at District Hospital Bergstrasse, Germany. All consecutive CEAs performed at District Hospital Bergstrasse from November 2013 to November 2017 were retrospectively reviewed. The surgical reports, anesthesia protocols, and medical records of the included patients were assessed. Cases were excluded if LRA was performed by an anesthesiologist experienced in LRA for CEA. The study was approved by the local ethics committee of the medical faculty at the University of Heidelberg, Germany (reference number: S-297/2017). Information that could identify individual patients was anonymized after data collection.

The primary endpoint of this study was the incidence of insufficient block quality from LRA, indicated by restlessness and pain (patient discomfort) or the need for additional analgesic medication during the procedure. Restlessness and pain represent relatively subjective parameters, both in the perception of patients and anesthesiologists. However, there is currently no generally accepted and more objective definition for regional anesthesia failure. The definition used in this document is based on a recommendation by Bottomley et al., recently published for peripheral nerve block failure [9]. Secondary endpoints were complications associated with LRA (e.g., Horner’s syndrome, dysphagia) and the rate of intraoperative conversion to general anesthesia due to block failure. For this purpose, intraoperative recordings in anesthesiologic and surgical records were retrospectively analyzed. In particular, corresponding notes from anesthesiologists and surgeons, vital signs, additional pain medication administered during the procedure, and records from the post-anesthesia care unit were considered. Furthermore, postoperative complications were examined using physician notes in discharge summaries and follow-up records. Upon individual patient request, premedication with midazolam was administered prior to LRA.

### 2.2. Intervention

In this study, the learning curves of nine board-certified anesthesiologists were assessed to evaluate the learnability of ultrasound-guided combined superficial and deep cervical plexus blockades for CEA. All anesthesiologists were already familiar with regional anesthesia techniques, both with and without ultrasound guidance, in other anatomical regions, but none had prior experience with LRA for CEA. Initial theoretical training and practical supervision included the following:

- Introduction to the fundamentals of ultrasound-guided combined superficial and deep cervical plexus anesthesia, including the anatomy of the cervical plexus, techniques for visualizing the target anatomical structures using ultrasound, needle guidance during the blockades, the selection and dosage of local anesthetics, and potential complications, all through a two-hour theoretical training session.
- The first six ultrasound-guided cervical plexus blockades performed by each anesthesiologist were conducted under the supervision of an anesthesiologist with prior experience in this form of regional anesthesia for CEA to demonstrate practical feasibility.
- Additionally, some of the participating anesthesiologists independently chose to attend external training sessions to further enhance their proficiency in the procedure.

As recommended for cervical plexus anesthesia, 40 mL of 0.5% ropivacaine (Ropivacaine Hydrochloride 0.5%, Fresenius Kabi AG, Bad Homburg, Germany) was routinely administered, with 20 mL targeting the deep cervical plexus and 20 mL targeting the superficial cervical plexus [10,11,12]. For anesthesia of the deep cervical plexus, as recommended, the transverse processes of the C2 to C4 vertebrae were visualized using ultrasound, with the carotid bifurcation serving as an additional landmark. The anesthetic was deposited approximately 1 cm caudal to the bifurcation to ensure effective blockade of the deep cervical plexus branches. The needle was advanced in-plane under continuous ultrasound guidance toward the target area near the C3 transverse process to maintain precise visualization and maximize patient safety [13,14]. For anesthesia of the superficial cervical plexus, the posterior border of the sternocleidomastoid muscle was visualized using ultrasound in the region of the lateral cervical triangle. The goal was to infiltrate along the posterior border of the sternocleidomastoid muscle in a cranio-caudal direction to achieve anesthesia at the nerve point (punctum nervosum). The needle was guided in-plane to provide the highest possible level of patient safety [13,15].

In patients under LRA, neurological status was continuously monitored during the procedure through communication with them. Additionally, patients were instructed to squeeze a squeaky ball with the hand on the contralateral side of the operative area every 10 s during the clamping phase. If any new neurological symptoms occurred during the clamping phase, arterial shunting of the operative area was performed. The mean arterial blood pressure was kept above 100 mmHg during the clamping phase. After declamping, a systolic blood pressure below 140 mmHg was targeted. To maintain hemodynamics within the desired range, both during and after the procedure, urapidil hydrochloride, clonidine hydrochloride, norepinephrine, and Akrinor^®^ were administered as needed. In cases of insufficient analgesia from the cervical plexus blockades, local anesthetics were administered by the surgeons and/or systemic anesthetics by the anesthesiologists, respectively, indicating block failure. If analgesia remained insufficient for the patient, conversion to GA was performed. In cases of general anesthesia, an arterial shunt was routinely placed intraoperatively. If LRA was contraindicated or rejected by the patient, GA was primarily performed with the same blood pressure targets. Technical neuromonitoring or cerebral oximetry were not available, which made LRA the preferred anesthesia technique for CEA in this setting.

### 2.3. Statistical Methods

The patients were divided into groups based on the experience of the attending anesthesiologist, with Group A including those who received LRA from anesthesiologists with experience in ≤ 3 cervical plexus blockades, Group B from those performing their 4th to 6th blockades, Group C from those performing their 7th to 9th blockades, and Group D from those with experience in more than 9 blockades. Groups A, B, and C were formed to represent three consecutive plexus blockades performed by each anesthesiologist. This number has proven sufficient in other studies to capture initial learning progress and was therefore adopted for our study [16]. Group D includes all LRA performed after an estimated learning phase of 9 blockades and is intended to represent the complication rate among experienced anesthesiologists, which remains above zero [9]. Baseline characteristics, block failure rates, and the frequency of anesthesia-associated complications were analyzed descriptively. Subsequently, statistical analysis was performed using the Pearson chi-square test for independence or Fisher’s exact test if the sample size was smaller than five. To avoid a Type I error, the significance level was adjusted using the Bonferroni correction [17]. Findings were considered statistically significant if the *p*-value was <0.05 or <0.0083 after Bonferroni correction. Finally, the results were graphically presented using a dual-axis chart, displaying the cumulative number of LRA procedures performed alongside the continuously decreasing cumulative block failure rate.

The software used for data collection and analysis included Microsoft Excel (Microsoft Corporation, Redmond, WA, USA) and IBM SPSS Statistics 24 (IBM Corporation, Armonk, NY, USA).

## 3. Results

### 3.1. Baseline Characteristics of the Cohort

Over the 48-month study period, a total of 126 CEAs were performed at District Hospital Bergstrasse, Germany, and retrospectively analyzed. Thirty-eight cases were excluded because perioperative care was managed by an anesthesiologist experienced in locoregional anesthesia techniques for carotid surgery. In the remaining 88 cases, the attending anesthesiologists had no prior experience with these anesthesia techniques. These patients formed the cohort for our study. Of these, 83 CEAs were primarily performed under LRA and five under GA. Further epidemiological characteristics of the cohort are provided in Table 1.

### 3.2. Complications During Locoregional Anesthesia for CEA

Insufficient block quality, indicated by restlessness and pain, or the requirement for additional intraoperative analgesic medication (locally administered by the surgeons or intravenously by the anesthesiologists, respectively), occurred in 15 patients. Three of these fifteen patients required conversion to GA during the procedure due to ongoing insufficient analgesia. Two patients reported dysphagia, while another developed Horner’s syndrome following the cervical plexus blockade. Additionally, five patients experienced nausea during the surgical procedure (see Table 2).

### 3.3. Complication Rate in Relation to the Number of Previously Performed LRA Procedures

The patients were divided into groups (A, B, C, and D) based on the experience of the attending anesthesiologist. Group A included those who received LRA from anesthesiologists with experience in ≤ 3 cervical plexus blockades, Group B from those performing their 4th to 6th blockades, Group C from those performing their 7th to 9th blockades, and Group D from those with experience in 10 or more blockades. Group A included 21 patients, Group B included 12 patients, Group C included 9 patients, and Group D included 41 patients (see Table 3). As mentioned above, cervical plexus blockades for patients in Groups A and B were performed under the supervision of an anesthesiologist experienced in this technique. LRA for patients in Groups C and D was performed without supervision, with patients in Group C representing the first three independently performed blockades by anesthesiologists who had newly learned the LRA technique.

When considering the frequency of complications in relation to the number of LRA procedures performed, the following was observed:

Insufficient block quality, indicated by restlessness and pain or the need for additional analgesic medication, occurred most frequently during the first three LRA procedures performed by each anesthesiologist (Group A; see Table 3). The statistical analysis revealed significant differences in the incidence of insufficient block quality among the groups (*p* = 0.003), with Group A showing the highest incidence (42.9%; see Table 3). After three LRA procedures, the incidence of block failure was significantly reduced (Group A vs. B, *p* = 0.008; see Table 3). Furthermore, the incidence of insufficient block quality did not increase again in Group C, representing the first three independently performed LRA procedures by each anesthesiologist after the supervision period.

**Figure 1:**
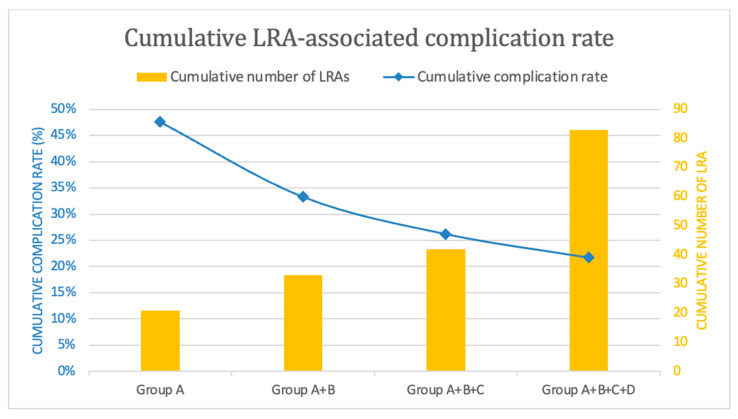
Locoregional anesthesia-associated complication rate.

In contrast, Group D showed a renewed increase in the incidence of insufficient block quality compared to Groups B and C. However, in relative terms, the increase was small and did not reach statistical significance. Correspondingly, a continuous overall decrease in the incidence of complications with the cumulative number of cervical plexus blockades performed was observed, as shown in Figure 1 (see Figure 1).

The conversion rate from LRA to general anesthesia was highest in Group A, where the attending anesthesiologist had the least experience with the technique. Horner’s syndrome and dysphagia, as additional complications related to locoregional anesthesia, occurred statistically independently of the number of previously performed LRA procedures (see Table 3).

Figure 1 illustrates the cumulative LRA-associated complication rate in our cohort plotted against the number of LRA procedures performed, categorized by the study groups A–D on a dual-axis chart. The *x*-axis represents the groups (A–D). The primary *y*-axis shows the cumulative complication rate as a line graph, while the secondary *y*-axis displays the cumulative number of LRA procedures as a bar graph. The complications considered in this illustration include insufficient block quality, dyspnea, dysphagia, and Horner’s syndrome. Conversions to general anesthesia occurred due to insufficient block quality and are not counted additionally.

## 4. Discussion

In this study, we demonstrated, in 83 patients, that ultrasound-guided LRA (combined deep and superficial cervical plexus anesthesia) for CEA can be rapidly mastered by board-certified anesthesiologists. The overall complication rates were low, with insufficient block quality being the most frequent complication (n = 15, 18.1%), leading to conversions to GA in three cases. After three LRA procedures performed under supervision, we observed a significant reduction (*p* = 0.008) in the incidence of block failure, which did not increase during the first unsupervised blockades. Additional complications related to the locoregional anesthesia technique included dysphagia (n = 2) and Horner’s syndrome (n = 1). However, the incidence of complications decreased continuously with the cumulative number of plexus blockades performed.

Our data, suggesting the rapid mastery of newly learned LRA under ultrasound guidance, are consistent with other findings. Helayel et al. demonstrated that, in inexperienced residents, only six practical exams were necessary after a theoretical introduction to reliably identify all structures of the axillary part of the brachial plexus using ultrasound, which forms the foundation of ultrasound-guided regional anesthesia [18]. In a phantom model of a peripheral nerve block, Kim et al. trained inexperienced medical students in hand–eye coordination, another key competence for safe and successful LRA. They found that five subsequent trials were sufficient to significantly improve hand–eye coordination for promising LRA outcomes [19]. These findings demonstrate the good learnability of key competencies for ultrasound-guided LRA, even for completely inexperienced examiners. In clinical practice, the application of these competencies may be complicated by factors such as anatomical variability in the target region and physician–patient interaction, which could increase the number of procedures needed for successful implementation [20,21]. Consistent with this, Morros et al. found that, in clinical scenarios, anesthesiologists with experience in nerve stimulator-guided regional anesthesia could successfully perform an independent ultrasound-guided blockade of the axillary plexus after 15 self-conducted blocks [22]. When compared to other procedures in anesthesiology, such as intubation—where approximately 200 intubations are required for safe mastery—ultrasound-guided LRA appears to be a rapidly learnable procedure [23]. In various studies, both nerve stimulator-guided and ultrasound-guided LRA have achieved favorable success rates, with ultrasound-guided techniques proving quicker to learn [8,24,25]. In line with these findings, we observed a rapid reduction in the incidence of insufficient block quality for cervical plexus blockades after only three supervised cervical plexus blockades performed by board-certified anesthesiologists with experience in LRA in other regions. This highlights the good learnability and transferability of ultrasound-guided LRA, particularly for the cervical plexus region, for anesthesiologists experienced in locoregional anesthesia techniques.

Following the rapid reduction in the incidence of block failure and other complications related to LRA in our cohort, we observed an interesting, though statistically non-significant, increase in incidence in Group D. Since this renewed increase in insufficient block quality did not occur in Group C (representing patients receiving the first three independently performed LRA procedures without supervision), the level of experience does not seem to provide a plausible explanation for this rise. Rather, the larger number of patients in Group D seems to offer a more accurate reflection of the average block failure and complication rates associated with LRA techniques, which evidently persist even among experienced anesthesiologists for various reasons [9,15,26,27]. This is further supported by the continuously decreasing overall complication rate we observed, calculated based on the cumulative number of LRA procedures performed.

In our cohort, no outcome-relevant complications associated with LRA were observed. Restlessness and pain, indicative of insufficient block quality, were the most frequent complications, occurring in 18.1% of patients and leading to conversions to GA in 3.6% of cases. Similarly, other studies report conversion rates to GA ranging from 0% to 4.3% [1,28,29,30,31,32,33]. As in our cohort, pain and restlessness were the most frequent causes of intraoperative conversions to GA [10,28]. Rates of insufficiency or failure of LRA, requiring supplementary intraoperative injections of local or systemic anesthetics, have been reported to range widely from 3% to 54% [10,30,34]. Since restlessness and pain represent the relatively subjective perceptions of both the patient and the attending anesthesiologists, these large differences can be explained well. Additionally, the lack of a clear definition of ‘block failure’ makes it a difficult outcome parameter to compare [9]. The definition of block failure used in our study—restlessness and pain or the need for additional analgesic medication during the procedure—represents a relatively subjective criterion too. Various definitions of peripheral nerve block failure have been used in recent years, each lacking purely objective criteria [9,26]. In their recently published work, Bottomley et al. defined block failure as the inability to perform the planned procedure due to insufficient analgesia. They proposed, alongside subjective patient comfort perception, the additional administration of local or systemic analgesics [9]. This approach was also adopted in our study, with the understanding that the generalizability of the results is limited by this potential bias. However, the similar rates of block failure and conversions to GA reported in other studies underline the plausibility of our findings.

Further LRA-related complications observed in our study were dysphagia and Horner’s syndrome, likely due to accidental blockade of the stellate ganglion. In addition to these temporary complications, other studies report hemodynamic instability and respiratory insufficiency following the administration of local anesthetics in the cervical region, accidental puncture of vessels resulting in hematoma, or even intravascular injection of a local anesthetic, among others [15,35]. To reduce severe complications such as intravascular injections and vascular injuries, the ultrasound-guided technique has been shown to be superior to the neurostimulator-guided technique and is recommended by current guidelines [15,36]. Regarding different techniques for cervical plexus blockade, a systematic review of 69 observational studies found that superficial or intermediate blocks have the lowest risk of failure or complications and should therefore be the preferred techniques today [31]. These recommendations are supported by recent findings from Opperer et al., who demonstrated that the deep cervical plexus block, particularly due to its involvement of the phrenic nerve, negatively affects diaphragm motion and thus may pose a potential risk for respiratory impairment in patients with pre-existing pulmonary conditions [33]. Consequently, at our hospital, the superficial cervical plexus block has since been established as the standard technique for LRA in CEA. But even with these techniques, a certain complication rate persists among experienced LRA experts due to various reasons [9,26,27,37]. In particular, the significant interindividual anatomical variability and interconnections within the cervical plexus inherently seem to preclude a universally ‘optimal application site’ with 100% efficiency [15,20,21]. Furthermore, the inconsistent nomenclature of the cervical region and its fasciae in relation to the cervical plexus complicates the cross-study comparison of complication rates [38]. However, the overall complication rate of 22% and failure rate of 18% observed in our study align with reports in the literature on failure and complication rates, interestingly, despite the use of different cervical plexus block depths (superficial, intermediate, and deep) in various studies [9,26,37,39]. The cause of this appears to lie in the unique anatomy of the cervical fasciae, which allow good permeability for injected fluids [33,40]. Thus, the exact spread and, consequently, the specific blockade site of the cervical plexus can only be predicted to a limited extent by a specific injection depth. Consistent with this, clinically relevant complications appear with the same frequency even when explicitly comparing different block depths [33]. Therefore, it is primarily the ultrasound-guided technique, rather than the block depth of the plexus, that positively influences safety and success rates and is now considered the standard.

Looking at LRA from the patient’s perspective, high satisfaction is evident. According to Davies et al., 92% of patients would choose LRA again for a subsequent procedure [41]. It is not only patients who prefer this technique; a preference for LRA, particularly the ultrasound-guided method, has also been demonstrated among residents [42]. Various curricula for learning LRA techniques have now been established [43,44].

Our study had several limitations. Firstly, due to the retrospective and single-center design, there was no standardized perioperative documentation of complications, and our data were not externally validated. Additionally, the relatively small sample size limits the validity of our data. Therefore, the present study design does not allow for generalization of the results or application to other medical settings or populations. Furthermore, every retrospective data analysis carries an inherent risk of bias regarding data completeness. Secondly, only board-certified anesthesiologists experienced in local anesthesia techniques were involved, which makes our results only partially transferable to other physicians. Due to the retrospective analysis, differences in group sizes (A-D) arose, as not every anesthesiologist performed the same number of LRA procedures. This may have obscured any effects of individual variations in learning curves. Thirdly, we used restlessness and pain as indicators of insufficient LRA, which are relatively subjective parameters. Additionally, preoperative anxiety influences pain levels and has been shown to be an independent risk factor for LRA failure [45,46]. Finally, the significant anatomical variation in the target region, such as interindividual sensory innervation by cranial nerves and peripheral nerves of the cervical plexus, contributes to varying individual responsiveness to LRA [15,20,21]. Therefore, prospective, multi-center studies with a larger number of participants and procedures are desirable to more accurately capture the learning curve of LRA techniques among anesthesiologists, as only such a design would allow for the generalization of the results, which is not feasible in our study. Additionally, a clear definition of ‘insufficiency/failure of LRA’ as an outcome parameter would be helpful to allow for better comparison between studies. Thus, our study should primarily be seen as an example and idea for conducting a suitably designed study that can generate transferable findings and be used to develop a curriculum.

## 5. Conclusions

Our study highlights the rapid learnability of ultrasound-guided cervical plexus blockades for board-certified anesthesiologists experienced in ultrasound-guided anesthesia techniques, demonstrating a low overall risk and supporting the training of anesthesiologists in this technique. It appears to require limited training effort in this specific setting, although no absolute conclusions can be drawn about the number of procedures needed to achieve proficiency due to the study design. However, this approach effectively integrates the advantages of ultrasound-guided LRA for CEA into clinical practice. While there appears to be a clear anesthesiologic advantage to LRA for CEA, the choice of anesthesia technique ultimately remains an interdisciplinary decision involving the entire treatment team.

## Figures and Tables

**Table 1:** **Baseline characteristics of the cohort:** baseline characteristics (numbers and mean values) of all patients who were managed by anesthesiologists inexperienced in anesthesia techniques for carotid endarterectomy.

	Frequences
Total number of patients [n]	88
Age [years], mean (range)	71.9 (52; 86)
ASA classification, mean (range)	2.9 (2; 4)
Sex: male [n]	60 (68.2%)
Sex: female [n]	28 (31.8%)
Asymptomatic stenosis of carotid artery [n]	54 (61.4%)
Symptomatic stenosis of carotid artery [n]	34 (38.6%)
Premedication [n]	17 (19.3%)
Duration of procedure [min], mean (range)	107.3 (61; 181)
Eversion CEA [n]	38 (43.2%)
Conventional CEA [n]	50 (56.8%)
Primary intraoperative shunt [n]	18 (20.5%)
Secondary intraoperative shunt [n]	4 (4.5%)
Clamping time [min], mean (range)	26.0 (1; 59)
Primary locoregional anesthesia [n]	83 (94.3%)
Primary general anesthesia [n]	5 (5.7%)
Akrinor^®^ [mL], mean (range)	1.3 (0; 5)
Norepinephrine [µg], mean (range)	0.0 (0; 200)
Clonidin [µg], mean (range)	62.2 (0;300)
Urapidil [mg], mean (range)	13.0 (0;150)

ASA: American Society of Anesthesiology; CEA: carotid endarterectomy; µg: microgram; ml: milliliters; min: minutes; n: number.

**Table 2:** **Complications during LRA for CEA:** incidence of complications associated with locoregional anesthesia during carotid endarterectomy.

	Incidence of Complications [n]
Total number of patients with LRA	83
Insufficient block quality (restlessness and pain)	15 (18.1%)
Intraoperative conversion to general anesthesia	3 (3.6%)
Dyspnea	0
Nausea	5 (6.0%)
Horner’s syndrome	1 (1.2%)
Dysphagia	2 (2.4%)

CEA: carotid endarterectomy; LRA: locoregional anesthesia; n: number.

**Table 3:** **Statistical analysis of the incidence of insufficient block quality and LRA-associated complications:** incidence of insufficient block quality and LRA-associated complications across the groups and results of the statistical analysis, which revealed significant differences in the incidence of block failure. A significance level of *p* < 0.05, or *p* < 0.0083 after Bonferroni correction, was applied.

	Group A (≤3 LRA)	Group B (4–6 LRA)	Group C (7–9 LRA)	Group D (≥10 LRA)	Chi-Square (*p*-Value)	Post Hoc A vs. B (*p*-Value)	Post Hoc A vs. C (*p*-Value)	Post Hoc A vs. D (*p*-Value)
Number of patients [n]	21	12	9	41				
Number of anesthesiologists [n]	9	4	4	3				
Incidence of block failure [n]	42.9% [9]	0% [0]	0% [0]	14.6% [6]	0.003	0.008	0.019	0.014
Incidence of intraop. conversions to GA [n]	9.5% [2]	0% [0]	0% [0]	2.4% [1]	0.383			
Incidence of Horner´s syndrome [n]	0% [0]	8.3% [1]	0% [0]	0% [0]	0.112			
Incidence of dysphagia [n]	4.8% [1]	0% [0]	0% [0]	2.4% [1]	0.798			

GA: general anesthesia; intraop: intraoperative; LRA: locoregional anesthesia; n: number; vs.: versus.

## Data Availability

The original contributions presented in the study are included in the article; further inquiries can be directed to the corresponding author.
